# The persistence and ecological impacts of a cyanobacterium genetically engineered to express mosquitocidal *Bacillus thuringiensis* toxins

**DOI:** 10.1186/s13071-016-1544-z

**Published:** 2016-05-10

**Authors:** Irene Ketseoglou, Gustav Bouwer

**Affiliations:** School of Molecular and Cell Biology, University of the Witwatersrand, , Private Bag 3, Wits, 2050 Johannesburg, South Africa

**Keywords:** *Anabaena* PCC 7120, *Bacillus thuringiensis israelensis*, *Anopheles arabiensis*, Genetically modified organism, Cry proteins, Microcosms, Non-target organisms

## Abstract

**Background:**

The cyanobacterium *Anabaena* PCC 7120#11 has been genetically engineered to act as a delivery vehicle for *Bacillus thuringiensis* subspecies *israelensis* mosquitocidal toxins. To address ecological concerns about releasing this genetically engineered microorganism into the environment for mosquito larva control, the persistence and ecological impacts of PCC 7120#11 was evaluated using multi-species, standardized aquatic microcosms.

**Methods:**

The microcosms were set up as described in ASTM E1366-02 (Standard Practice for Standardized Aquatic Microcosms: Fresh Water), with a few modifications. The treatment group microcosms were inoculated with PCC 7120#11 and key water quality parameters and non-target effects were compared between the treatment and control groups over a period of 35 days.

**Results:**

PCC 7120#11 decreased from a concentration of 4.50 × 10^6^ cells/ml (at inoculation) to 1.32 × 10^3^ cells/ml after 4 weeks and larvicidal activity against third instar larvae of *Anopheles arabiensis* was only evident for two weeks after treatment. Both treatment and the interaction of treatment and time had a significant effect on nitrate, phosphate and photosynthetic microorganism concentrations. Treatment with PCC 7120#11 caused a temporary spike in ammonia in the microcosms a week after treatment, but the concentrations were well below acute and chronic criteria values for ammonia in freshwater ecosystems. *Cyprinotus vidua* concentrations were not significantly different between PCC 7120#11 and control microcosms. In PCC 7120#11 microcosms, *Daphnia pulex* concentrations were significantly lower than control concentrations between days 18 and 25. By the end of the experiment, none of the measured variables were significantly different between the treatment groups.

**Conclusions:**

The standard aquatic microcosm experiments provided more data on the ecological impacts of PCC 7120#11 than single-organism assessments would have. On the basis of the relatively minor, short-term effects that PCC 7120#11 had on water quality parameters and non-target invertebrates, further evaluation of PCC 7120#11 for use in integrated vector management is warranted.

## Background

*Bacillus thuringiensis* subspecies *israelensis* (*Bti*) is a spore-forming, aerobic, gram-positive bacterium that produces crystalline inclusions that contain Cry (crystal) or Cyt (cytolytic) proteins that are toxic to nematoceran larvae [1]. Cry and Cyt proteins are pore-forming toxins that act on the midgut epithelial cells [2].

Mosquitoes play a major role in the transmission of malaria and other diseases, such as the Zika virus disease [3, 4]. Biological control offers an environmentally low-impact method for the control of mosquito larvae and provides another tool for use in integrated vector management (IVM), which is becoming increasingly reliant on successfully managing insecticide resistance [5]. Although *Bti* and formulations of *Bti* have been moderately successful as vector control agents (e.g. [6, 7]), they do not exhibit long-term larvicidal activity in the environment and may thus require frequent treatments for sustained vector control [8–10]. In an attempt to overcome the limitations of *Bti* as a larvicidal agent, microorganisms have been genetically engineered with the aim of producing *Bti*-based control agents that persist longer in the environment [8, 11].

The cyanobacterium, *Anabaena* sp. strain PCC 7120 (hereafter referred to as PCC 7120), is a filamentous, nitrogen-fixing cyanobacterium capable of multiplying in mosquito oviposition sites and maintaining its position in the water column [8, 9], thereby making it a suitable candidate for genetic engineering for use as a mosquito control agent. Furthermore, unlike some species of cyanobacteria, PCC 7120 is not considered toxic [12], and is unlikely to affect humans or animals. As a result of its suitable characteristics, PCC 7120 was genetically engineered to act as a delivery vehicle for *Bti* toxins. The *cry4Aa*, *cry11Aa*, and *p20* genes from *Bti* were introduced into PCC 7120 using an *Escherichia coli*-*Anabaena* shuttle vector pRL488p [13]. The resultant clone, PCC 7120#11, has been shown in laboratory assays to be highly larvicidal to *Aedes aegypti* [9, 13], and several important malaria-carrying vectors such as *Anopheles gambiae*, *Anopheles arabiensis*, and *Anopheles merus* [14, 15]. The *Bti* genes, which are under the control of two tandem promoters (cyanobacterial constitutive promoter, *P*_*psbA*_, and *E. coli* T7 early promoter, *P*_*A1*_), are integrated into the chromosome of PCC 7120#11, resulting in a stable recombinant strain [15].

Although response surface methodology experiments have shown that PCC 7120#11 can be efficiently produced in photobioreactors [16], there are potential risks with large-scale releases of genetically engineered microorganisms (GEMs) in the field [8, 17, 18]. Due to the concerns of testing PCC 7120#11 in the field and the difficulty in obtaining regulatory approval for field trials of GEMs, laboratory aquatic microcosms are the most feasible method of testing GEMs that are ultimately intended for application to natural waterbodies. Of particular interest in this study was the use of a multi-species system, the standardized aquatic microcosm (SAM), to evaluate the persistence and ecological impacts of PCC 7120#11.

The SAM method is a simple and reliable method that mimics a generalized aquatic community, with important ecological processes, such as primary and secondary production, being demonstrated [19, 20]. The microcosms are standardized in that the experiment is initiated with the same chemically defined medium and sediment, same species assemblage and concentration of invertebrate species and photosynthetic microorganisms (PMs) [20, 21]. This allows for repeatability within a laboratory and between laboratories [21]. Although SAM experiments are more complex than single-species tests, they are not as complex as natural environments and thus reduce the likelihood that taxonomic uncertainties will occur [21]. Another advantage of the SAM protocol is that the experiments can be conducted and initiated at any time of the year [21].

As part of an assessment of the usefulness of PCC 7120#11 in IVM, we evaluated the effects of PCC 7120#11 on a target organism (*An. arabiensis*) and ecotoxicology test organisms using the SAM system. *Anopheles arabiensis* was selected as the target organism since it is the most widespread vector of malaria in southern Africa [22]. Cladocerans, ostracods, and rotifers are useful and important aquatic ecotoxicology test organisms [23–25], and the SAMs used in this study included representatives of each of these metazoan groups. Since the metazoans in the SAMs are able to ingest filamentous cyanobacteria (e.g. [26, 27]), the SAMs would facilitate detection of any direct toxicity mediated by the larvicidal *Bti* proteins expressed in PCC 7120#11. The SAMs also enable detection of indirect effects, such as changes in growth rates due to changes in the preferred food sources of an organism. To the best of our knowledge, this is the first study to assess the persistence and ecological impacts of a genetically engineered cyanobacterium using SAMs.

## Methods

### Microcosms

The microcosms were set up as described in ASTM E1366-02 Standard Practice for Standardized Aquatic Microcosms: Fresh Water [28], with a few modifications. The SAMs were kept in a growth room that was set at 21 ± 1 °C. Cool white lights were used for illumination (4700 lx; approximately 63 μmol m^-2^ s^-1^ photosynthetic photon flux density) with a L:D 12:12 h photoperiod.

The microcosm setup is described briefly. The microcosms consisted of sterile glass jars (4 l) containing 3 l of autoclaved chemically defined medium (T82MV) [28]. Autoclaved silica sand enriched with chitin and cellulose powder was added to the glass jars. Six jars (replicates) were allocated for PCC 7120#11 and six jars (replicates) for the control, with an additional five prepared in case of breakages within the first week [28]. The glass jars were covered individually with square glass plates to minimize contamination and evaporation. Nine different species of photosynthetic microorganisms (PMs), which included algal and cyanobacterial species, were added to the jars on day 0 (start of the microcosms) and four species of invertebrates were added on day 4. After 7 days, quality control was performed to ensure the well-being of the communities in the microcosms [28]. On day 7, PCC 7120#11 was added to PCC 7120#11 microcosms to produce an in-microcosm PCC 7120#11 concentration of 4.5 × 10^6^ cells/ml. The organism abundance (PMs and invertebrates) and inorganic nutrient concentrations were measured twice a week.

### Culturing of PMs

*Anabaena cylindrica*, *Ankistrodesmus* sp., *Chlamydomonas reinhardtii*, *Chlorella vulgaris*, *Oscillatoria simplicissima*, *Scenedesmus obliquus*, *Raphidocelis subcapitata* (syn. *Selenastrum capricornutum*), *Stigeoclonium* sp., and *Ulothrix* sp., were purchased from the University of Texas (UT) Algal Culture Collection. The PMs were cultured under continuous illumination (2000 lx; approximately 27 μmol m^-2^ s^-1^ photosynthetic photon flux density) at 25 °C, in individual 1 l Erlenmeyer flasks containing sterile T82MV medium [20]. The cells were counted using a Palmer cell and were added to the microcosms at a concentration of 5 × 10^3^ cells/ml. Filamentous PMs or PMs that tended to form clumps were, prior to counting and before being added to the microcosms, vigorously shaken in sterile jars with sterile glass beads [28]. The concentrations were calculated using the equation presented in the ASTM E1366-02 protocol [28].

### Culturing of non-target invertebrates

*Daphnia pulex* (obtained from colonies at the Department of Water Affairs, Pretoria, South Africa) was cultured in sterile 3 l glass jars containing sterile hard water (123 mg/l MgSO_4_; 96 mg/l NaHCO_3_; 4.0 mg/l KCl; 60 mg/l CaSO_4_.2H_2_O). Daphnids were cultured at 18 °C with a L:D 18:6 h photoperiod and were fed a 1:1:1 mixture of yeast, fish food (TetraMin®), and alfalfa (Mpho Khumalo, pers. comm.). The cultures were fed three times a week and sub-cultured every 2 weeks to avoid over-population. Sixteen *D. pulex* (three adults, three adults with eggs, and ten small) were added to each of the microcosm jars on day 4 [28]. Although the standard SAM setup includes the amphipod crustacean *Hyalella azteca* [28], we did not include this organism in our SAMs because, in contrast to its status in North America, it is not an important crustacean in South Africa.

The ostracod, *Cypridopsis vidua* was cultured in sterile glass beakers (500 ml) containing sterile T82MV medium [20]. Ostracods were cultured at 18 °C with a L:D 18:6 h photoperiod and fed weekly with a mixture of *Stigeoclonium* sp. and *C. vulgaris* cultures [28]. On day 4 of the experiment, *C. vidua* were removed from the beakers, rinsed in sterile T82MV medium, and then added to each microcosm jar (six ostracods per jar) [28].

The protozoans (*Coleps* sp.) and the rotifers (*Lepadella* sp.) were cultured in Petri dishes containing T82MV medium, yeast, and *C. vulgaris*. They were cultured at 25 °C under L:D 12:12 h photoperiods. Three hundred protozoans and 90 rotifers were added to each microcosm jar on day 4 of the experiment [28].

### Mosquitocidal activity of PCC 7120#11

Third instar *An. arabiensis* larvae were obtained from colonies (origin: Kanyemba*,* Zimbabwe; colony name: KGB) maintained at the National Institute of Communicable Diseases (NICD) in Johannesburg, South Africa. The larvae were reared at the NICD in a controlled environment of 25 °C, relative humidity of 60 %, and a L:D 12:12 h photoperiod with a 45 min dawn and dusk light regime.

The larvicidal activity of PCC 7120#11 in the PCC 7120#11 microcosms was determined by the weekly (starting on day 7) addition of 30 third instar larvae of *An. arabiensis*. The mortality of the larvae was recorded 24 h post-inoculation (p.i.), after which all larvae (dead or alive) were removed from each treatment microcosm. Larvae were added also to the control (no PCC 7120#11) microcosms.

### Sample collection and analysis

Sampling and measurement of organism abundances and other variables were performed twice a week on the same days of the week (Tuesdays and Fridays). As part of quality control procedures, the pH of each microcosm was monitored using a HI 9828 multi-parameter HANNA probe (HANNA Instruments, Rhode Island, USA). After the pH measurements, the microcosms were mixed using a sterile spatula and the sides of the jars were scraped as completely as possible with a sterile rubber spatula [28]. Samples (20 ml) from the mixed microcosms were filtered through 0.45 μm (25 mm diameter) cellulose acetate syringe filters (Sartorius Biotech, Göttingen, Germany). At some sampling times, the clogging of filters necessitated the use of more than one syringe filter per 20 ml sample. The filtrates were analyzed for key water quality parameters using a Spectroquant® NOVA 60 photometer (Merck, Darmstadt, Germany) and photometric Spectroquant® test kits: NO_3_-N (kit 1.09713), NO_2_-N (kit 1.14776), PO_4_-P (kit 1.14848), and NH_4_-N (kit 1.14752; measures both ammonium ions and dissolved ammonia). Due to unforeseen circumstances, chemical analyses were not performed on day 32 of the experiments.

A sample (4 ml) was removed from each of the mixed microcosms with 2 ml being used for PM counts and 2 ml used for *Coleps* sp. and *Lepadella* sp*.* counts. The PM counts were performed using a Palmer cell, examining a maximum of 65 fields of view [28]. The rotifers and protozoans were counted in 100 μl aliquots in 96 well micro-titer plates, with a total of 2 ml being evaluated [28]. Aliquots (100 ml) of each stirred microcosm were removed (using a large-bore, dip sampler) for the *D. pulex* and *C. vidua* counts [28]. All aliquots were returned to the microcosms after the counting was performed.

### Statistical analysis

Data were analyzed using the Mixed Models – Repeated Measures procedure of NCSS 10 Statistical Software (NCSS, LLC. Kaysville, Utah, USA). A longitudinal design was used, with one between-subjects factor (treatment) and one within-subjects factor (time). The AR(1) pattern was used for the variance-covariance structure, with selection of this pattern being supported by Akaike information criterion (AIC) model fit assessments. Counts were log transformed [log_10_ (x + 1)], a transformation that is preferred when some of the observed values are small numbers or zero [29, 30]. The denominator degrees of freedom for *F* tests were computed using the Kenward and Roger adjustment [31], an adjustment proposed for small sample settings. When statistical analyses indicated that a factor or an interaction of factors had a significant effect on the response, data were analyzed further by *post-hoc* Bonferroni multiple comparison tests. Differences were considered significant at α = 0.05. Although treatment × time interactions may be considered most informative for this study, assessment of treatment as a main factor is useful for interpreting the results, especially in the absence of a significant treatment × time interaction for a response. The slopes of the growth curves of the metazoan organisms were analyzed using the linear regression model within the nonlinear regression module of GraphPad Prism version 6.05 for Windows (GraphPad Software, La Jolla, USA).

## Results

### Time as a factor

**Fig. 1:**
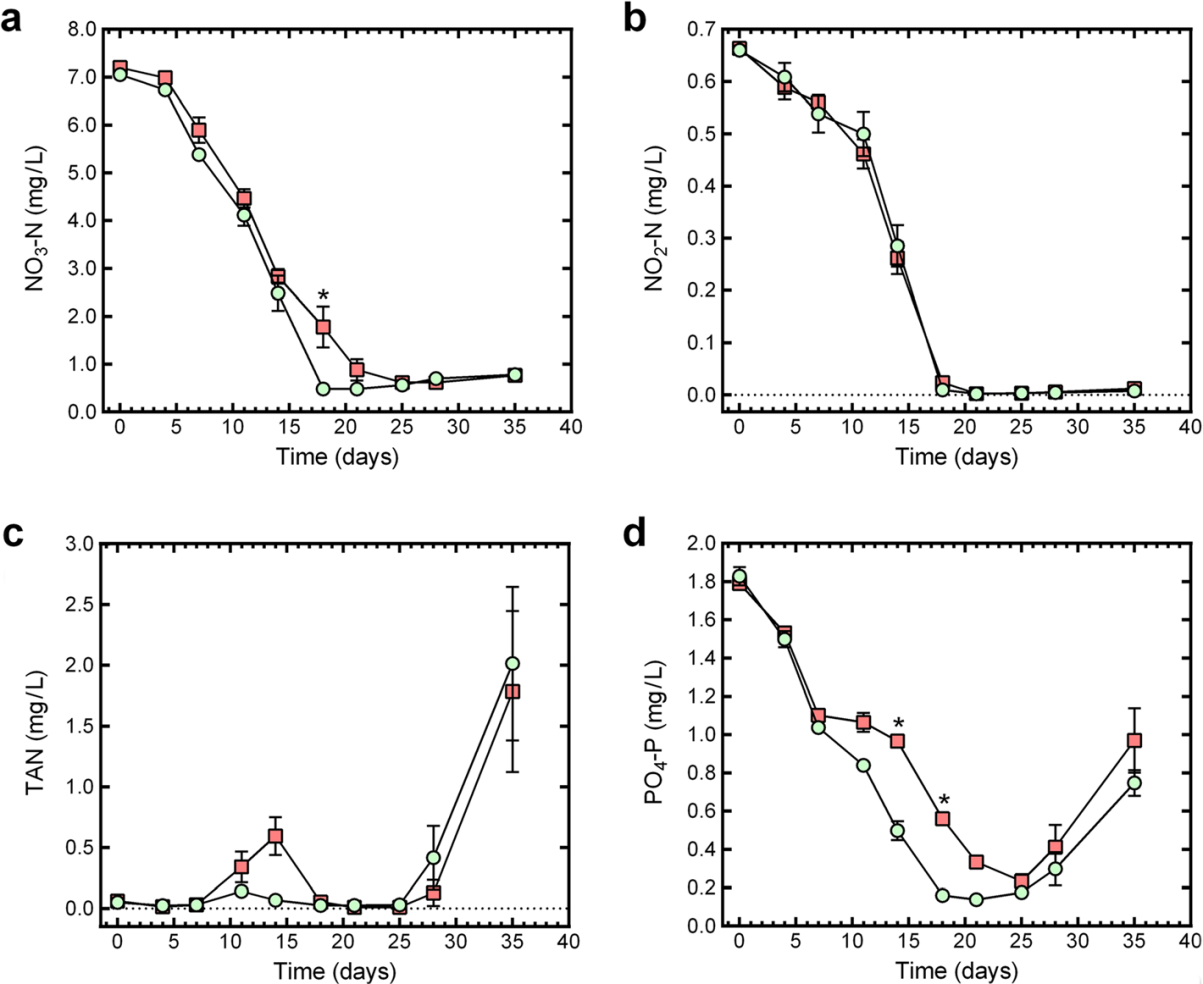
The change in nitrate, nitrite, ammonia and phosphate concentration in control (*light green circle*) and PCC 7120#11 (*red square*) microcosms during the 35-day evaluation period. **a** NO_3_-N; **b** NO_2_-N; **c** total ammonia nitrogen (TAN); and **d** PO_4_-P. Data points show the mean ± standard error of the mean, with an *asterisk* indicating a significant difference between the means of PCC 7120#11 and control microcosms on the evaluation day

**Fig. 2:**
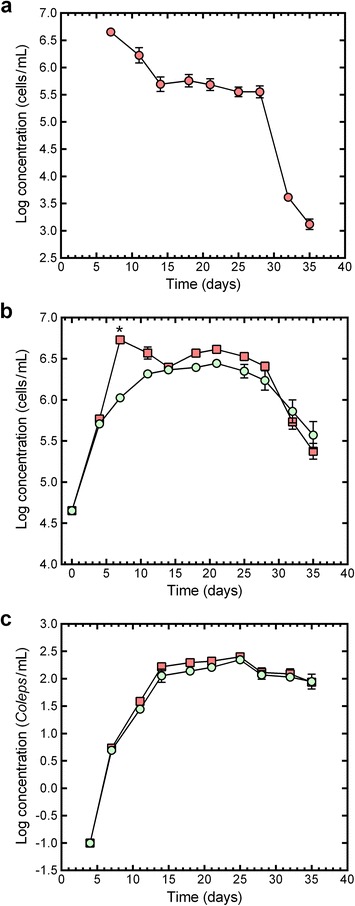
The change in the concentration of unicellular organisms in control (*light green circle*) and PCC 7120#11 (*red square*) microcosms during the 35-day evaluation period. **a** PCC 7120#11 concentration in the PCC 7120#11 microcosms (inoculation was on day 7); **b** total photosynthetic microorganisms; and **c** *Coleps* sp. (ciliate). Data points show the mean ± standard error of the mean, with an *asterisk* indicating a significant difference between the means of PCC 7120#11 and control microcosms on the evaluation day

**Fig. 3:**
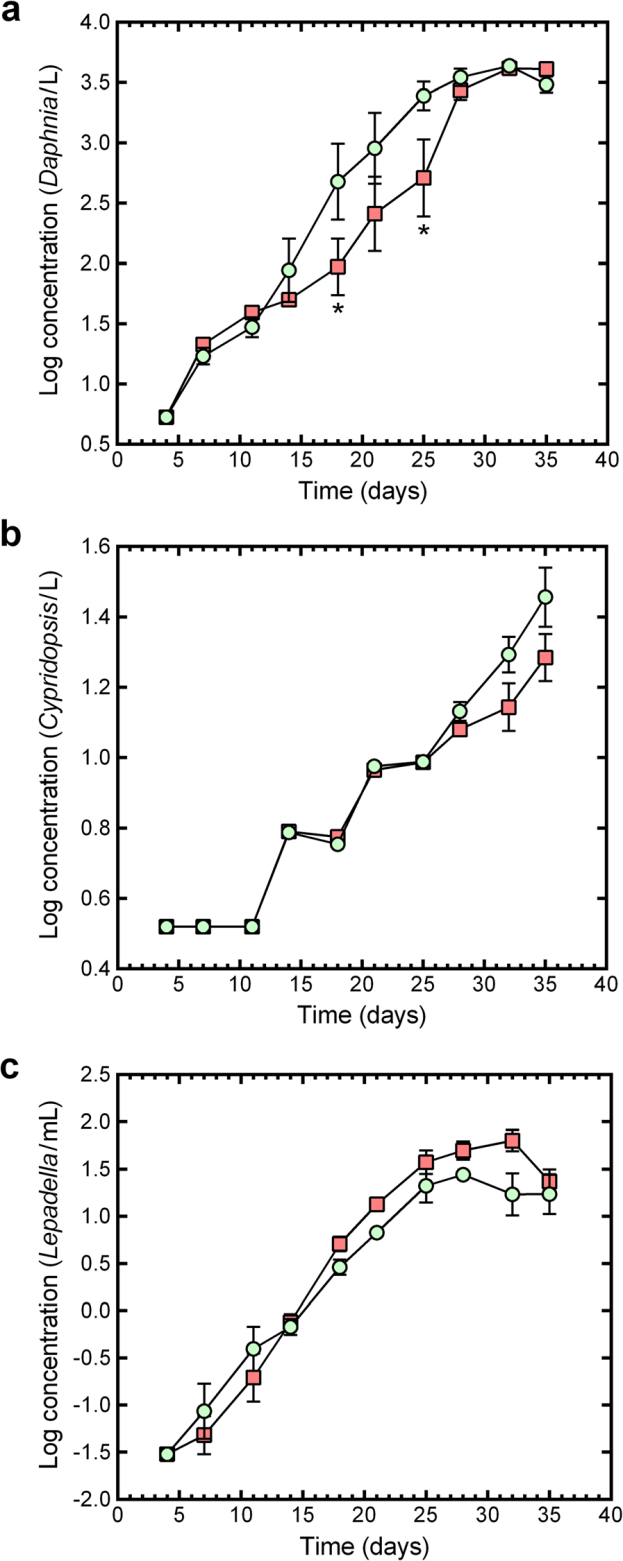
The change in the concentration of metazoan organisms in control (*light green circle*) and PCC 7120#11 (*red square*) microcosms during the 35-day evaluation period. **a** *Daphnia pulex* (cladoceran); **b** *Cypridopsis vidua* (ostracod); and **c** *Lepadella* sp. (rotifer). Data points show the mean ± standard error of the mean, with an *asterisk* indicating a significant difference between the means of PCC 7120#11 and control microcosms on the evaluation day

As expected, time had a significant effect (*P* < 0.0001) on each of the evaluated responses (water quality parameters and organism concentrations) and time as a main factor will not be discussed further. For each of the evaluated responses, the concentration curves (mean profile plots) for the control and PCC 7120#11 microcosms were similar in shape (Figs. 1, 2 and 3), but for some responses there were significant interactions of treatment (PCC 7120#11 or control) and time. These interactions are discussed in more detail below.

### Nitrogen compounds

Treatment, as a main factor, had a significant effect (*F*_(1, 36.1)_ = 15.3921, *P =* 0.0004) on nitrate (NO_3_-N) concentration, with the mean nitrate concentration being higher in the PCC 7120#11 microcosms (3.21 mg/l) than in the control microcosms (2.88 mg/l). The treatment × time interaction was significant (*F*_(9, 83.5)_ = 2.3985, *P =* 0.0180) for nitrate concentration, with the nitrate concentration being significantly higher (*P* < 0.0001) in the PCC 7120#11 microcosms than in the control microcosms on day 18 (Fig. 1a).

The nitrite (NO_2_-N) concentration decreased from an initial concentration of approximately 0.66 mg/l to below 0.025 mg/l by day 18 and remained below 0.025 mg/l until the end of the experiment in both microcosm types (Fig. 1b). The treatment effect was not significant for nitrite concentration (*F*_(1, 23.8)_ = 0.0816, *P =* 0.7777), and there was no significant treatment × time interaction for nitrite concentration (*F*_(9, 85.9)_ = 0.7794, *P =* 0.6358).

The initial concentration of total ammonia nitrogen (TAN: NH_4_-N + NH_3_-N) was approximately 0.05 mg/l in both microcosm types (Fig. 1c). After day 28, the concentration of TAN in the microcosms increased rapidly and reached concentrations of between 1.8 and 2.0 mg/l by day 35 (Fig. 1c). The treatment × time interaction was not significant for TAN concentration (*F*_(9, 66.6)_ = 0.4885, *P =* 0.8773), and there was no significant difference (*F*_(1, 5.2)_ = 0.0080, *P =* 0.9320) for TAN concentration between treatment groups.

### Phosphate

The phosphate (PO_4_-P) concentration decreased after initiation of the experiments in both microcosm types (Fig. 1d), but between days 25 and 35 the phosphate concentration in the microcosms increased (Fig. 1d). Treatment had a significant effect on phosphate concentration (*F*_(1, 8.9)_ = 7.8673, *P =* 0.0208), with the mean phosphate concentration being higher in the PCC 7120#11 microcosms (0.90 mg/l) than in the control microcosms (0.72 mg/l). The treatment × time interaction was also significant (*F*_(9, 79.4)_ = 3.6865, *P =* 0.0007) (Fig. 1d).

### Photosynthetic microorganisms

In the PCC 7120#11 microcosms, the PCC 7120#11 concentration decreased between days 7 and 14 but then remained relatively constant in the region of 4.5 × 10^5^ cells/ml for 14 days (from day 14 to day 28) (Fig. 2a). By day 35, the PCC 7120#11 concentration had decreased to approximately 1.32 × 10^3^ cells/ml (Fig. 2a).

The main effect of treatment was significant for PM concentration (*F*_(1, 13.2)_ = 4.9768, *P =* 0.0437), with the mean PM concentration being higher in the PCC 7120#11 microcosms (1.32 × 10^6^ cells/ml) than in the control microcosms (9.83 × 10^5^ cells/ml). The treatment × time interaction was significant for PM concentration (*F*_(10, 90.7)_ = 9.0738, *P <* 0.0001), with the mean PM concentration on day 7 (the day of inoculation with PCC 7120#11) in the PCC 7120#11 microcosms being significantly higher (*P* < 0.0001) than that in the control microcosms (Fig. 2b). From the high of approximately 5.36 × 10^6^ cells/ml on day 7, the PM concentration in the PCC 7120#11 microcosms decreased to approximately 2.48 × 10^6^ cells/ml by day 14 (Fig. 2b). After day 14, the PM concentration curves had comparable profiles in the control and PCC 7120#11 microcosms (Fig. 2b).

### Protozoans

In both treatment groups, *Coleps* sp. populations increased rapidly from day 4 until day 14, then increased gradually to a maximum of approximately 250 *Coleps*/mL by day 25 (Fig. 2c). The *Coleps* sp. concentrations in both microcosm types decreased gradually between days 25 and 35 (Fig. 2c). The treatment × time interaction was not significant for the *Coleps* sp. concentration (*F*_(9, 80.7)_ = 0.4025, *P =* 0.9303), but the main effect of treatment was significant for *Coleps* sp. concentration (*F*_(1, 25.2)_ = 4.6167, *P =* 0.0415), with the mean concentration being 51.3 *Coleps*/ml and 60.8 *Coleps*/ml for the control and PCC 7120#11 microcosms, respectively.

### Cladocerans

The *D. pulex* concentrations increased in an exponential-like manner in both microcosm types (Fig. 3a), but after day 11 the *D. pulex* populations in the control microcosms increased more rapidly than the *D. pulex* populations in the PCC 7120#11 microcosms (Fig. 3a). The treatment × time interaction was significant for *D. pulex* concentration (*F*_(9, 87.8)_ = 2.2878, *P =* 0.0234), with *post-hoc* tests showing that the mean *D. pulex* concentration in the PCC 7120#11 microcosms was significantly lower than that in the control microcosms on day 18 (*P =* 0.0244) and day 25 (*P =* 0.0280). The slope (0.098; adjusted *r*^*2*^ = 0.754) of the *D. pulex* growth curve for the PCC 7120#11 microcosms was significantly lower (*F*_(1, 92)_ = 4.621, *P =* 0.0342) than that of the control microcosms (slope = 0.122; adjusted *r*^*2*^ = 0.828) between day 4 and day 28 (the apparent exponential growth phase). By day 32, *D. pulex* populations in the two types of microcosms had peaked at comparable concentrations (Fig. 3a), and treatment (as a main factor) did not have a significant effect on the concentration of *D. pulex* (*F*_(1, 19.5)_ = 2.3399, *P =* 0.1421).

### Ostracods

The mean profile plots for *C. vidua* concentrations had similarly-shaped profiles in the control and PCC 7120#11 microcosms (Fig. 3b), with *C. vidua* populations exhibiting a lag phase from day 4 to day 11. The *C. vidua* population growth between days 11 and 25 was characterized by alternating periods of growth and no-growth plateaus (Fig. 3b). The treatment × time interaction was not significant for *C. vidua* concentration (*F*_(9, 75.6)_ = 1.3300, *P =* 0.2360), and treatment (as a main factor) also did not have a significant effect on *C. vidua* concentration (*F*_(1, 10.3)_ = 1.9715, *P =* 0.1896).

### Rotifers

For both treatment groups, *Lepadella* sp. growth was characterized by exponential-like growth between days 4 and 25 (Fig. 3c). The peak *Lepadella* sp. concentrations were obtained on day 28 and day 32 for the control and PCC 7120#11 microcosms, respectively (Fig. 3c). The treatment × time interaction was not significant for *Lepadella* sp. concentration (*F*_(9, 81.8)_ = 1.7985, *P =* 0.0809). There was a significant treatment effect for *Lepadella* sp. concentration (*F*_(1, 16.7)_ = 4.8487, *P =* 0.0421; means: control = 5.5 *Lepadella*/ml and PCC 7120#11 = 7.7 *Lepadella*/ml). The slope (0.159; adjusted *r*^*2*^ = 0.914) of the *Lepadella* sp. growth curve for the PCC 7120#11 microcosms was significantly higher (F_(1, 80)_ = 4.734, *P =* 0.0325) than that of the control microcosms (slope = 0.135; adjusted *r*^*2*^ = 0.864) between day 4 and day 25 (the apparent exponential growth phase).

### Mosquitocidal activity of PCC 7120#11

A single application of PCC 7120#11 was added to each of the PCC 7120#11 microcosms on day 7, at an in-microcosm concentration (4.5 × 10^6^ cells/ml) that, on the basis of pre-SAM bioassays, should have resulted in approximately 90 % mortality (i.e. an LC_90_) of the third instar *An. arabiensis* larvae. After the addition of PCC 7120#11, *An. arabiensis* larval mortality on day 8 was 72.3 ± 4.5 % (mean ± standard error of the mean; *n* = 180; 6 replicates of 30 larvae per replicate). A week later (day 15), larval mortality dropped to 17.7 ± 8.3 %. On the remaining evaluation days (22, 29, and 36), no mortality of the *An. arabiensis* larvae was observed. *An. arabiensis* larval mortality was not observed in any of the control microcosms.

## Discussion

In contrast to other larvicides, such as naturally-derived spinosad and the organophosphate temephos, *Bti* products have little effect on non-target aquatic organisms [32, 33]. The challenge with the assessment of the ecological impacts of PCC 7120#11 is that it is in essence an edible delivery vehicle for larvicidal *Bti* proteins and is a food source for several species of aquatic invertebrates. This means that ecotoxicological and impact assessments of PCC 7120#11 should ideally assess both the toxicity of the *Bti* proteins to important test organisms and the effects that a significant increase in a food source may have on the growth and reproduction of these test organisms. The SAM system enables the assessment of both the direct toxicity of PCC 7120#11 to important aquatic test organisms and potential indirect effects that PCC 7120#11 may have on the survival, growth and reproduction of the test organisms.

Since one of the advocated advantages of PCC 7120#11 is that it would multiply and persist in waterbodies and thus reduce the need for repeated treatments [8, 9], we performed these experiments using a single inoculation of PCC 7120#11 to produce an in-microcosm concentration of 4.5 × 10^6^ cells/ml. The concentration of 4.5 × 10^6^ cells/ml, which was determined by bioassays in SAMs to be an LC_90_, was 3.15-fold higher than the LC_90_ (1.43 × 10^6^ cells/ml) for *An. arabiensis* reported for 100-ml cup assays [14]. The lower LC_90_ in the cup assays compared to the SAM assays is not unexpected since larvae ingest only PCC 7120#11 in the cup assays, whereas in the SAMs the ingestion of other PMs and protozoans by the mosquito larvae would likely reduce the relative intake of PCC 7120#11 cells.

Not unexpectedly, because of the high number of PCC 7120#11 cells added to the PCC 7120#11 microcosms on day 7, the effect of treatment on total PM concentration was statistically significant and the concentration of PMs on day 7 was significantly higher in the PCC 7120#11 microcosms than in the control microcosms. However, since the concentration of PCC 7120#11 decreased rapidly after day 7, the concentration of PMs was not statistically different between the two treatment groups on any other day during the experiment. The decrease in the concentration of PCC 7120#11 between day 7 and day 14 was most probably due to predation of the cells by the invertebrate grazers.

The concentration of inorganic nutrients (nitrate, nitrite, and phosphate) in the control and PCC 7120#11 microcosms decreased from day 0 (initiation) and was largely depleted by day 20, as expected due to the utilization of these nutrients by the rapidly growing population of PMs [19, 20, 34, 35]. The depletion of the inorganic nutrients coincided with the highest concentration of PMs recorded in the microcosms. The changes in the inorganic nutrients are discussed further below in the context of the activities of the different organisms in the microcosms.

Aquatic PMs are efficient in removing phosphate from solution and most of this phosphate is released back into the water upon cell death [36]; e.g. 50 % of particulate phosphate is released into the water as PO_4_-P within a few hours of the onset of autolysis [37]. Since the “sloppy feeding” of grazers (especially daphnids) on PMs damage cells and result in the loss of intracellular components [38], we would expect large amounts of PO_4_-P to be released when invertebrate grazers feed on PMs in the microcosms. In this context, the significantly higher PO_4_-P concentrations observed in the PCC 7120#11 microcosms after the addition of PCC 7120#11 to the microcosms may reflect the release of PO_4_-P from the feeding of the invertebrate grazers on the high concentration of PCC 7120#11 cells.

Being an excreted waste product, the level of ammonia is expected to increase as the number of invertebrates increase [35], and NH_3_ has been shown to be the predominant released form of nitrogen for *D. pulex* [39]. However, although the numbers of invertebrates increased in both the control and PCC 7120#11 microcosms throughout the experiments, the TAN concentrations only started to increase markedly in the microcosms after day 25. The TAN concentrations in the microcosms is likely to be the result of the complex interaction between several processes, including excretion of ammonia by the metazoan consumers [39], and PM-mediated processes such as assimilatory nitrate reduction and dinitrogen fixation by diazotrophic cyanobacteria (including PCC 7120#11). Since ammonia is preferentially used as an inorganic nitrogen sources by many algae and most cyanobacteria [40, 41], dissolved ammonia would be taken up by PMs and there would be significant intracellular ammonia pools in the PMs [41].

There are two ways that the ammonia could be released by PMs: during growth or due to damage or lysis of cells. Cyanobacteria, and in particular *Anabaena* spp., have been shown to release ammonia (NH_3_), both in the presence and absence of nitrate in the medium, during different growth phases [40]. Since the ammonia spike (days 11 and 14) in the PCC 7120#11 microcosms corresponds with the rapid decrease in PCC 7120#11 cells between days 7 and 14, we postulate that the ammonia spike is primarily due to the release of ammonia from PCC 7120#11 cells due to “sloppy feeding” by invertebrate grazers. Support for this “sloppy feeding” postulate is provided by the fact that the increase in ammonia in both treatment groups after day 25 corresponds with a rapid and consistent reduction in PMs between days 25 and 35. Since ammonia is a preferred nitrogen source for PMs, we would expect them to rapidly utilize the “extra” ammonia that was available in the PCC 7120#11 microcosms. The utilization of the “extra” ammonia is suggested by the temporary nature of the day-14 ammonia peak in the PCC 7120#11 microcosms. This increased availability of ammonia may also have been, in part, responsible for the slight (not statistically significant) increase, relative to the control microcosms, in PM concentrations for approximately two weeks after the day-14 ammonia spike in the PCC 7120#11 microcosms.

Ammonia is known to be highly toxic to aquatic invertebrates [42], and may have significant ecological sub-lethal effects even at low concentrations. Although the TAN concentration was not statistically higher in the PCC7120#11 microcosms than in the control microcosms, it is important to determine if the TAN levels reached theoretical toxic levels during the temporary, post-inoculation increase in TAN. When considering the most sensitive endpoints, the 2013 Ambient Water Quality Criteria (AWQC) for ammonia document specifies an acute (1-hour average) criterion magnitude and a chronic (30-day rolling average) criterion magnitude for freshwater ecosystems [42]. Since NH_3_ is substantially more toxic than NH_4_^+^ and the ratio of NH_3_ to NH_4_^+^ increases as the pH and temperature of the water increases [43], we used the pH and temperature corrected AWQC to determine if the ammonia levels during the spike on day 14 (pH 7.5 and 22 °C) in the PCC 7120#11 microcosms would potentially be toxic to highly-sensitive aquatic organisms. However, the post-inoculation TAN spike (0.595 mg/l on day 14) in the PCC 7120#11 microcosms was well below the pH- and temperature-adjusted values of 7.8 and 1.2 mg TAN/l for the acute criterion and chronic criterion magnitudes, respectively. Although our experiments suggest that treatment of waterbodies with PCC 7120#11 would not result in toxic levels of NH_3_, further studies using a range of pH and temperatures would be useful.

The statistically higher nitrate concentration on day 18 in the PCC 7120#11 microcosms may also reflect the availability of a preferred nitrogen source (ammonia) during the two measurement periods preceding day 18; i.e. the PMs may have utilized ammonia instead of nitrate, thus resulting in a higher level of nitrate in the PCC 7120#11 microcosms than in the control microcosms (which did not have the ammonia spike).

The SAM experiments showed that the larvicidal activity of PCC 7120#11 did not persist in the microcosms after the single inoculation. By day 14, the PCC 7120#11 concentration was 4.92 × 10^5^ cells/ml, which is 9.14-fold lower than the concentration at inoculation (4.5 × 10^6^ cells/ml). Since PCC 7120#11 is capable of multiplying in mosquito breeding sites [8, 9], the relatively poor persistence of larvicidal activity observed in this study was most likely due to the inability of PCC 7120#11 to replicate in the microcosms at a rate that matched or exceeded their removal by other organisms in the microcosms. In this context, it is important to note that all three types of the metazoan consumers in the microcosms were able to ingest filamentous cyanobacteria (e.g. [26, 27]). The fairly constant concentration of PCC 7120#11 between days 14 and 28 even though there were logarithmic increases in the non-target invertebrates may, in part, be because PCC 7120#11 is a filamentous cyanobacterium and the smaller, unicellular algae, such as *C. vulgaris,* may be more readily consumed by the invertebrates used in this study [44]. The marked reduction in larvicidal activity between days 7 and 14 may not only reflect the reduction by close to a factor of 10 in PCC 7120#11 concentration during this period but may also be due to the rapid and marked (30.5-fold) increase in the concentration of protozoans (a food source for mosquito larvae) during this period, which may have resulted in fewer PCC 7120#11 cells being ingested by mosquito larvae. In this light, it is also important to note that there were marked changes in the PCC 7120#11 microcosms in the ratio of the concentrations of total PMs to PCC 7120#11 cells between days 7 and 35: day 7, 1.19; day 14, 5.04; day 21, 8.49; day 28, 7.16; day 35, 180.4 (ratios are based on the antilog values of the data presented in Fig. 2a and b). Since the persistence of the larvicidal activity of *Bti*-based products are affected by several bioenvironmental factors [10], it is reasonable to expect that the persistence of the activity of PCC 7120#11 would also vary in different habitats. Further research is required to help elucidate the factors that affect PCC 7120#11 persistence in complex and dynamic aquatic ecosystems which contain a range of non-target invertebrates and particulate food sources.

Although *Coleps* spp. feed on bacteria and algae [45], the mode of action of larvicidal *Bti* proteins means that direct toxic effects of PCC 7120#11 on *Coleps* spp. are not expected. The population growth profiles of the *Coleps* sp. were characterized by a rapid increase between days 4 and 14 and a subsequent relatively stable plateau period. The onset of the plateau period corresponds approximately with the depletion of the inorganic nutrients, suggesting that the plateau period is due to nutrient-limiting conditions. Although rotifers and cladocerans (including *Daphnia* spp.) are predators of protozoans and can suppress ciliates [46, 47], there was no obvious reduction in *Coleps* sp. populations in either of the treatment groups during the period that *D. pulex* and *Lepadella* sp. concentrations increased. This suggests that *Coleps* sp. was either not a preferred prey or that the rate of predation did not exceed the rate of reproduction during the plateau phase.

Since PCC 7120#11 was present at more than 3.57 × 10^5^ cells/ml until day 28, the non-target organisms in the microcosms were exposed to a high PCC 7120#11 concentration for a period that matched that of the 21-day *D. magna* reproduction test [48]. *D. pulex* concentrations in the PCC 7120#11 microcosms were significantly lower than those in the control microcosms on days 18 and 25 and the slope of the *D. pulex* growth curve for the PCC 7120#11 microcosms was significantly lower than that of the control microcosms between days 14 and 28 (the last measurement before the apparent onset of the stationary phase). However, by day 32 the mean *D. pulex* concentration in the PCC 7120#11 microcosms was statistically identical to that in the control microcosms and when treatment effects were averaged over time there was no statistical difference in mean *D. pulex* concentration between the two treatment groups. This suggests that PCC 7120#11 temporarily lowered *D. pulex* growth rates, but with growth recovering and there being no long-term effects on *D. pulex* concentrations. The temporarily lowered growth rate may reflect a direct, sub-lethal effect of PCC 7120#11 on *D. pulex*, but more likely it reflects an indirect effect, such as a sub-lethal effect of the ammonia spike in the PCC 7120#11 microcosms on the growth of *D. pulex*.

There are other ways that PCC 7120#11 may have reduced the growth rate of *D. pulex*. Large daphnids, such *D. pulex*, can consume a large range of food sources, including small bacteria, ciliates and rotifers [46], and there were thus multiple potential food sources in the microcosms. However, since *Daphnia* spp. prefer the smaller, unicellular algae such as *C. vulgaris* [44], it is possible that PCC 7120#11 is an sub-optimal food source for *D. pulex* and has lower nutritional value than the standard SAM PMs, with the result that *D. pulex* individuals in the PCC 7120#11 microcosms were unable to match the growth rates of individuals in the control microcosms.

There was no evidence that PCC 7120#11 caused lethal toxicity to the *Lepadella* sp. in the PCC 7120#11 microcosms. This may not only be due to a lack of toxicity of larvicidal *Bti* proteins to the *Lepadella* sp. used, but may reflect the fact that rotifers ingest filamentous *Anabaena* spp. with low efficiency [26], thus possibly preventing exposure to lethal concentrations. The slope of the *Lepadella* sp. growth curve in the PCC 7120#11 microcosms between days 4 and 25 was significantly higher than that for the control microcosms and from day 14 onwards the *Lepadella* sp. concentrations in the PCC 7120#11 microcosms were higher than those in the control microcosms. Since day 14 corresponds to the post-inoculation spike of ammonia, and to the point at which the *D. pulex* concentrations in the PCC 7120#11 microcosms start to be lower than those in the control microcosms, it is possible that the higher concentrations of *Lepadella* sp. in the PCC 7120#11 microcosms were due to less suppression of *Lepadella* sp. by *D. pulex. Daphnia* spp. have been shown to suppress rotifers by exploitative competition for shared, limited food resources or by mechanical interference (damage upon attempted ingestion or ingestion) [26].

One of the advantages of PCC 7120#11 over *Bti* is that it is able to replicate and persist better in the environment [9], thus providing longer mosquito control and reducing the need for frequent retreatment of waterbodies. From an ecological and regulatory perspective, it would be preferable if the concentration of PCC 7120#11 reduces after inoculation and if PCC 7120#11 did not become a well-established member of the PM community in treated waterbodies. In this regard, it is noteworthy that in a period of 4 weeks, PCC 7120#11 decreased from a concentration of 4.50 × 10^6^ cells/ml (at inoculation) to 1.32 × 10^3^ cells/ml, a more than 3400-fold reduction.

## Conclusions

In this study, PCC 7120#11 caused small, temporary changes in selected water quality parameters and invertebrate concentrations, but the population growth curves for the non-target invertebrates were surprisingly similar for the two treatment groups. Although short-lived environmental events may have delayed and lasting effects [49], the small, temporary changes observed in the PCC 7120#11 microcosms should be seen in the context of commonly used mosquito control agents, which are known to be lethal to aquatic invertebrates and significantly affect aquatic diversity [32, 50]. When considering the relatively minor ecological impacts noted in these experiments and the high larvicidal activity of PCC 7120#11 against important malaria vectors [14, 15], we believe that further evaluation of PCC 7120#11 as a mosquito larvicide for use in integrated vector management is warranted.
